# A Web-Based Prediction Model for Cancer-Specific Survival of Elderly Patients Undergoing Surgery With Prostate Cancer: A Population-Based Study

**DOI:** 10.3389/fpubh.2022.935521

**Published:** 2022-07-12

**Authors:** Zhaoxia Zhang, Chenghao Zhanghuang, Jinkui Wang, Tao Mi, Jiayan Liu, Xiaomao Tian, Liming Jin, Dawei He

**Affiliations:** ^1^Department of Urology, Chongqing Key Laboratory of Children Urogenital Development and Tissue Engineering, Chongqing, China; ^2^Chongqing Key Laboratory of Pediatrics, Chongqing, China; ^3^Ministry of Education Key Laboratory of Child Development and Disorders, Chongqing, China; ^4^National Clinical Research Center for Child Health and Disorders, Chongqing, China; ^5^China International Science and Technology Cooperation Base of Child Development and Critical Disorders, Chongqing, China; ^6^Chongqing Higher Institution Engineering Research Center of Children's Medical Big Data Intelligent Application, Children's Hospital of Chongqing Medical University, Chongqing, China

**Keywords:** nomogram, SEER, prostate cancer, surgery, elderly, CSS

## Abstract

**Objective:**

Prostate cancer (PC) is the second leading cause of cancer death in men in the United States after lung cancer in global incidence. Elderly male patients over 65 years old account for more than 60% of PC patients, and the impact of surgical treatment on the prognosis of PC patients is controversial. Moreover, there are currently no predictive models that can predict the prognosis of elderly PC patients undergoing surgical treatment. Therefore, we aimed to construct a new nomogram to predict cancer-specific survival (CSS) in elderly PC patients undergoing surgical treatment.

**Methods:**

Data for surgically treated PC patients aged 65 years and older were obtained from the Surveillance, Epidemiology, and End Results (SEER) database. Univariate and multivariate Cox regression models were used to identify independent risk factors for elderly PC patients undergoing surgical treatment. A nomogram of elderly PC patients undergoing surgical treatment was developed based on the multivariate Cox regression model. The consistency index (C-index), the area under the subject operating characteristic curve (AUC), and the calibration curve were used to test the accuracy and discrimination of the predictive model. Decision curve analysis (DCA) was used to examine the potential clinical value of this model.

**Results:**

A total of 44,975 elderly PC patients undergoing surgery in 2010–2018 were randomly assigned to the training set (*N* = 31705) and validation set (*N* = 13270). the training set was used for nomogram development and the validation set was used for internal validation. Univariate and multivariate Cox regression model analysis showed that age, marriage, TNM stage, surgical style, chemotherapy, radiotherapy, Gleason score(GS), and prostate-specific antigen(PSA) were independent risk factors for CSS in elderly PC patients undergoing surgical treatment. The C index of the training set and validation indices are 0.911(95%CI: 0.899–0.923) and 0.913(95%CI: 0.893–0.933), respectively, indicating that the nomogram has a good discrimination ability. The AUC and the calibration curves also show good accuracy and discriminability.

**Conclusions:**

To our knowledge, our nomogram is the first predictive model for elderly PC patients undergoing surgical treatment, filling the gap in current predictive models for this PC patient population. Our data comes from the SEER database, which is trustworthy and reliable. Moreover, our model has been internally validated in the validation set using the C-index,AUC and the and the calibration curve, showed that the model have good accuracy and reliability, which can help clinicians and patients make better clinical decision-making. Moreover, the DCA results show that our nomogram has a better potential clinical application value than the TNM staging system.

## Background

The global incidence of prostate cancer (PC) is second only to lung cancer, and it is also the second leading cause of cancer death among men in the United States (1). PC is a heterogeneous disease with a variable natural history (2). With the introduction of serum prostate-specific antigen(PSA) testing in the late 1980s, the age at diagnosis of PC was nearly 10 years earlier, so surgeons can now provide more prompt surgical intervention early in tumor development without waiting for advanced stage (3).

PC has a variety of treatment options, including active surveillance (AS), surgical resection, radiotherapy (RT), chemotherapy, local ablation therapy (cryotherapy, high-intensity focused ultrasound), etc. Surgery is the primary treatment modality for PC, including radical prostatectomy (RP) and local tumor resection. However, while up to half of the patients with PC require only active surveillance at initial diagnosis, most men are willing to undergo aggressive local surgical treatment. Radical prostatectomy is associated with improved cancer-specific mortality(CSM), metastasis-free survival, and the need for palliative care, and surgical resection may be more beneficial for PC patients than radiotherapy (4). Xia et al. demonstrated that men undergoing radical prostatectomy had an increased life expectancy by 1.8 months compared to active surveillance (5). The above studies suggest that patients with PC treated with surgery may have a better prognosis. However, some studies suggest that surgery does not have much benefit in the prognosis of PC patients. The ProtecT trial reported the results of several extensive cohort studies suggesting that active surveillance could be safely used in carefully selected low-risk PC patients (6, 7). Elisabeth M et al. also found that surgical resection and radiotherapy did not benefit much from survival in patients with low-risk PC (4). We found that the current study is controversial regarding the impact of surgical treatment on the prognosis of PC patients. Based on this, we are eager to develop a model that can predict prognostic factors in PC patients undergoing surgery.

The traditional tumor-node-metastasis (TNM) cancer staging system of the American Joint Committee on Cancer (AJCC) has been used as a prognostic criterion for the vast majority of solid tumors. However, it does not include many important factors related to PC survival, such as age, prostate-specific antigen, Gleason score(GS), surgical, chemoradiotherapy, etc. In recent years, the nomogram prediction model has been considered one of the most accurate ways to predict tumors (8). Currently, nomograms based on the SEER database have been increasingly used to predict the prognosis of PC patients. Joao Ricardo Alves et al. constructed a nomogram predicting extracapsular extension in prostate cancer patients (9). In addition, researchers have developed nomograms that can predict lymph node metastases in patients with intermediate-risk prostate cancer, bone metastases in patients with newly diagnosed prostate cancer, survival in patients with prostate cancer spinal metastases, and survival in patients with metastatic prostate cancer undergoing radical prostatectomy (10–13). The incidence of PC is closely related to age. Data report that older men over 65 years old account for more than 60% of PC patients (14), and more than 90% of PC cancer-specific deaths (CSS) occur in this age group (15). Therefore, finding prognostic factors associated with CSS in surgically treated elderly PC patients has important clinical implications for reducing patient mortality. Unfortunately, however, to our knowledge, no nomogram has been developed for elderly PC patients undergoing surgical treatment. Therefore, our study aimed to use data from the SEER database to develop a nomogram for elderly PC patients undergoing surgical treatment that can accurately predict the relevant factors affecting patients' CSS to better assist clinicians and patients in making clinically aided decisions.

## Patients and Methods

### Data Source and Data Extraction

Data from patients diagnosed with PC between 2010 and 2018 were extracted from the SEER database, and our study targeted patients aged 65 years and older and undergoing surgical treatment. The SEER database is a national cancer database covering approximately 30% of the population and containing 18 cancer medical centers. No ethical approval and patient informed consent is required because the SEER database data are publicly available and hidden patient information. The research methodology used in this study follows the research guidelines published in the SEER database.

**Figure 1:**
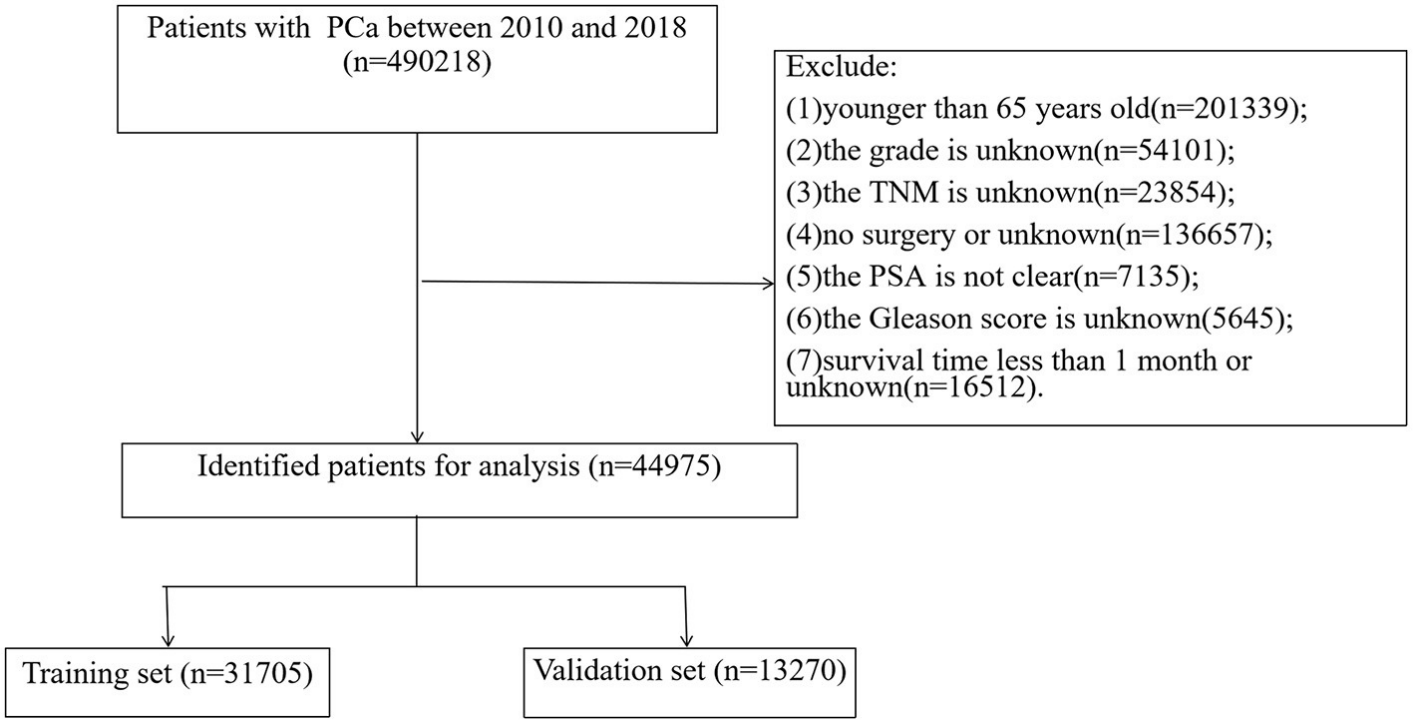
Flowchart for inclusion and exclusion of elderly PC patients undergoing surgery treatment.

The variables in the SEER database include demographic characteristics [age, race, marital status), tumor grade (grade I, II, III, and IV)], TNM stage, surgical method (local tumor resection, radical prostatectomy), radiotherapy, chemotherapy, prostate-specific antigen, Gleason score, survival status, cause of death and survival time. Patient ethnicity was classified as white, black, and other types. Inclusion criteria: (1) patients aged 65 years and older, (2) a pathological diagnosis of PC, and (3) patients undergoing surgical treatment. Exclusion criteria: (1) patients younger than 65 years old, (2) tumor grade is unknown, (3) TNM stage is unknown, (4) no surgical treatment or surgical method is unknown, (5) prostate-specific antigen is unclear, (6) Gleason score is unknown, (7) survival time is <1 month or survival time is unknown. The flowchart of patient inclusion and exclusion is shown in Figure 1.

### Development and Validation of the Nomogram

All patients were randomized into the training set (70%) and the validation set (30%) for nomogram development and internal validation. Univariate and multivariate Cox proportional regression models were used to identify independent risk factors for patients in the training set. The nomogram was established based on the restivariate Cox regression analysis, and results were used to predict CSS at 3-, 5-, and 8-year in surgically treated elderly PC patients. In addition, the nomograms were calibrated at 3-, 5-, and 8-year, and these assessments used 1,000 autonomous samples. The accuracy and discrimination of the model were tested by the consistency index (c-index) and the area under the subject operating characteristic curve (AUC).

### Clinical Application

The decision analysis curve (DCA) is used to assess the clinical value of the nomogram. We also calculated the risk for each patient from the nomogram. All patients were divided into high-risk and low-risk groups using the cutoff value of the subject operating characteristic curve (ROC). The Log-rank test and the Kaplan-Meier (K-M) curves examined the differences in survival between high-risk and low-risk patients. In addition, we analyzed differences between surgery and radiotherapy in high-risk and low-risk groups.

### Statistical Analysis

Means and standard deviations were used to describe continuous variables such as age. Chi-square or non-parametric U tests were used for comparison between groups. Other classifications were described by frequency (%), and the differences between the groups were compared using the chi-square test. The Cox regression model analyzed the patient prognostic factors, and the patient survival differences were analyzed by the log-rank test and the K-M curve. Statistical analyses were performed using the R software version 4.1.0 and SPSS26.0.The R package includes "survival,” “ggDCA,” “DynNom,” and “RMS” *P* values < 0.05 were considered statistically significant.

## Results

### Clinical Features

A total of 44,975 PC patients from 2010 to 2018 were included in our study from the SEER database who were all over 65 years old and were treated surgically, including the training set (*N* = 31705) and the validation set (*N* = 13270). The mean age of all patients was 70.0 ± 4.73 years, and most patients were white (82.7%) and married (76%). The tumor grade was mainly at grade II (42.2%) and grade III (48.9%). The T stage was dominated by T2 (48.6%), followed by T1 (22.6%) and T3 (26.5%), and minimal T4 (2.38%). All patients were mainly N0 (95.1%) and M0 (98.3%). Most patients underwent radical prostatectomy (83.2%), and fewer patients underwent local tumor resection (16.8%). Only 0.5% of the patients received chemotherapy. The majority of the patients (90.6%) did not receive radiotherapy. The Gleason score was mainly 7 (50.6%), and no significant difference between patients with Gleason scores ≤ 6 (24.2%) and Gleason score≥8 (25.2%). 73.6% patients with prostate-specific antigen <10 ng/ml, 17.8% with prostate-specific antigen 10–20 ng / ml and only 8.58% with prostate-specific antigen> 20 ng/ml.There was no significant statistical deviation in the clinical characteristics of both groups, and the results are shown in Table 1.

**Table 1:** Clinicopathological characteristics of elderly PC patients recieved surgery.

	**All**	**Training cohort**	**Validation cohort**	
	***N* = 44975**	***N* = 31705**	***N* = 13270**	**p**
Age	70.0 (4.73)	70.0 (4.73)	69.9 (4.74)	0.847
Race:				0.469
White	37215 (82.7%)	26192 (82.6%)	11023 (83.1%)	
Black	4016 (8.93%)	2861 (9.02%)	1155 (8.70%)	
Other	3744 (8.32%)	2652 (8.36%)	1092 (8.23%)	
Marital:				0.385
No/Unknown	10813 (24.0%)	7659 (24.2%)	3154 (23.8%)	
Married	34162 (76.0%)	24046 (75.8%)	10116 (76.2%)	
Grade:				0.765
I	3236 (7.20%)	2281 (7.19%)	955 (7.20%)	
II	19001 (42.2%)	13402 (42.3%)	5599 (42.2%)	
III	22003 (48.9%)	15517 (48.9%)	6486 (48.9%)	
IV	735 (1.63%)	505 (1.59%)	230 (1.73%)	
T:				0.893
T1	10153 (22.6%)	7184 (22.7%)	2969 (22.4%)	
T2	21838 (48.6%)	15380 (48.5%)	6458 (48.7%)	
T3	11913 (26.5%)	8393 (26.5%)	3520 (26.5%)	
T4	1071 (2.38%)	748 (2.36%)	323 (2.43%)	
N:				0.538
N0	42781 (95.1%)	30145 (95.1%)	12636 (95.2%)	
N1	2194 (4.88%)	1560 (4.92%)	634 (4.78%)	
M:				0.040
M0	44201 (98.3%)	31133 (98.2%)	13068 (98.5%)	
M1	774 (1.72%)	572 (1.80%)	202 (1.52%)	
Surgery:				0.375
Local tumor excision	7543 (16.8%)	5350 (16.9%)	2193 (16.5%)	
Radical prostatectomy	37432 (83.2%)	26355 (83.1%)	11077 (83.5%)	
Chemotherapy:				0.514
No/unknown	44748 (99.5%)	31540 (99.5%)	13208 (99.5%)	
Yes	227 (0.50%)	165 (0.52%)	62 (0.47%)	
Radiation:				0.813
No/unknown	40756 (90.6%)	28738 (90.6%)	12018 (90.6%)	
Yes	4219 (9.38%)	2967 (9.36%)	1252 (9.43%)	
Gleason:				0.716
≤ 6	10901 (24.2%)	7666 (24.2%)	3235 (24.4%)	
7	22736 (50.6%)	16013 (50.5%)	6723 (50.7%)	
≥8	11338 (25.2%)	8026 (25.3%)	3312 (25.0%)	
PSA:				0.644
<10	33122 (73.6%)	23338 (73.6%)	9784 (73.7%)	
10–20	7994 (17.8%)	5622 (17.7%)	2372 (17.9%)	
>20	3859 (8.58%)	2745 (8.66%)	1114 (8.39%)	
CSS:				0.117
Dead	1203 (2.67%)	873 (2.75%)	330 (2.49%)	
Alive	43772 (97.3%)	30832 (97.2%)	12940 (97.5%)	
Survival months	45.6 (31.2)	45.4 (31.2)	46.0 (31.3)	0.115

### COX Regression Analysis

A univariate Cox regression model was used to analyze and screen influencing factors associated with survival in the training set. The results showed that age, marriage, grade Grade III, TNM stage, surgical method, chemotherapy, radiotherapy, prostate-specific antigen, and Gleason score were the prognostic factors affecting patient survival. Then, a multivariate Cox regression analysis was used to screen for independent risk factors associated with patient survival. The results showed that age, marriage, TNM stage, surgical method, radiotherapy, chemotherapy, prostate-specific antigen, and Gleason score were independent risk factors affecting patient CSS. All of the results are shown in Table 2.

**Table 2:** Univariate and multivariate analyses of CSS in training cohort.

	**Univariate**				**Multivariate**		
	**HR**	**95%CI**	**P**		**HR**	**95%CI**	**P**
Age	1.18	1.17–1.19	<0.001		1.079	1.072–1.086	<0.001
**Race**							
White							
Black	1.1	0.87–1.39	0.43				
Other	0.88	0.68–1.13	0.307				
**Marital**							
No/unknown							
Married	0.55	0.48–0.63	<0.001		0.76	0.7–0.824	<0.001
**Grade**							
I							
II	0.86	0.5–1.48	0.586				
III	4.67	2.8–7.79	<0.001				
IV	2.65	0.6–11.6	0.197				
**T**							
T1							
T2	0.16	0.13–0.19	<0.001		0.856	0.762–0.961	0.009
T3	0.32	0.27–0.39	<0.001		1.042	0.899–1.208	0.585
T4	2.98	2.46–3.61	<0.001		1.581	1.343–1.86	<0.001
**N**							
N0							
N1	5.31	4.48–6.29	<0.001		1.393	1.211–1.603	<0.001
**M**							
M0							
M1	43.65	37.49–50.84	<0.001		2.597	2.243–3.008	<0.001
**Surgery**							
Local tumor excision							
Radical nephrectomy	0.09	0.08–0.1	<0.001		2.597	2.243–3.008	<0.001
**Chemotherapy**							
No/Unknown							
Yes	13.54	9.99–18.36	<0.001		1.723	1.294–2.296	<0.001
**Radiation**							
No/Unknown							
Yes	2.4	2.03–2.84	<0.001		0.797	0.709–0.897	<0.001
**PSA**							
<10							
10–20	2.28	1.9–2.73	<0.001		1.196	1.081–1.323	<0.001
>20	11.37	9.8–13.21	<0.001		1.356	1.211–1.519	<0.001
**Gleason**							
≤ 6							
7	1.97	1.44–2.69	<0.001		1.204	1.079–1.343	0.001
≥8	17.53	13.21–23.27	<0.001		2.048	1.822–2.302	<0.001

### Development and Validation of the Nomograms

**Figure 2:**
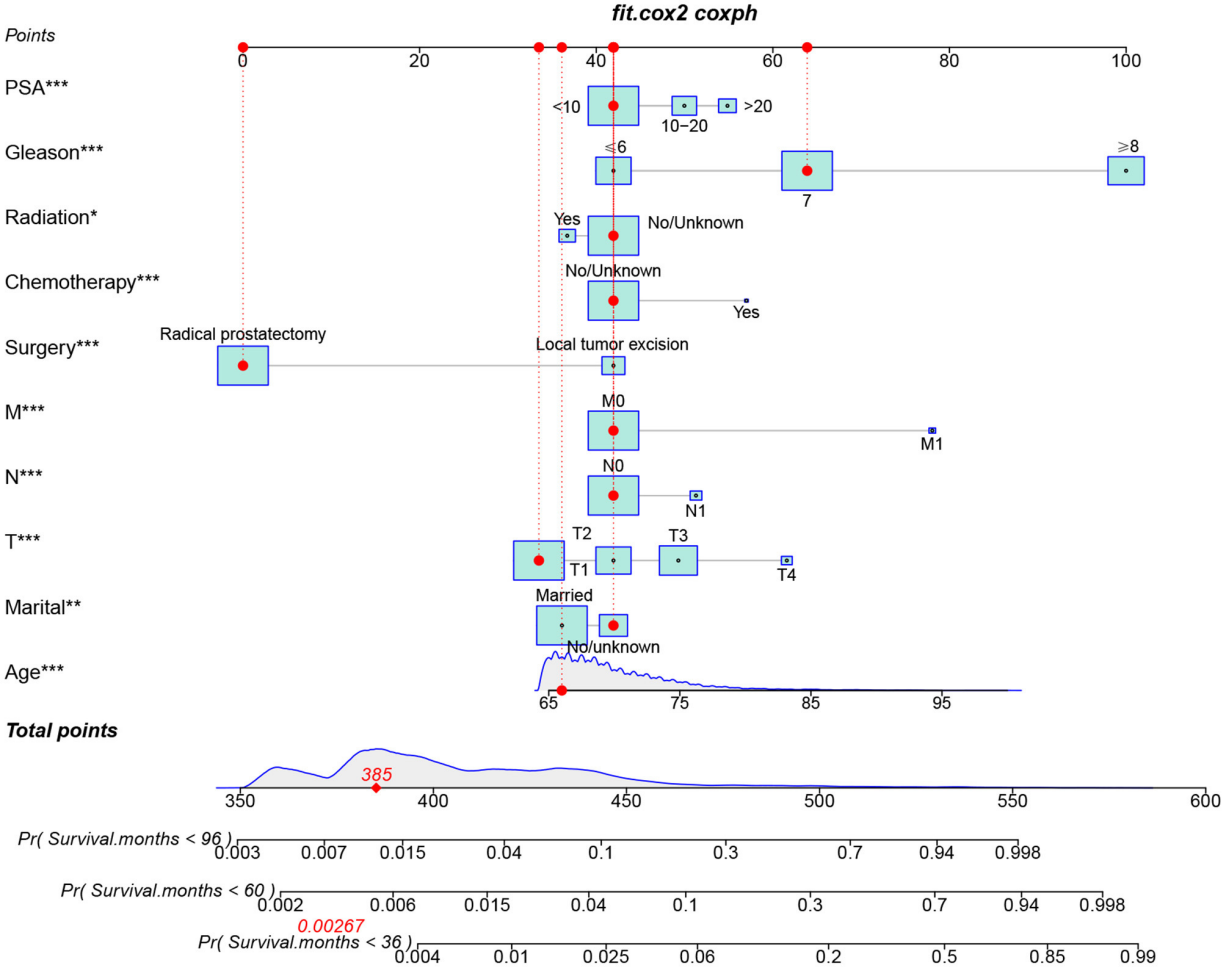
The nomogram for predicting 3-, 5-, and 8-year CSS in elderly PC patients undergoing surgery treatment.

We constructed a feasible nomogram based on a multivariable Cox regression analysis model to predict CSS at 3-, 5-, and 8-year in elderly PC patients undergoing surgery (Figure 2). Age, TNM stage, surgical method, prostate-specific antigen, and Gleason score are the most significant factors influencing CSS in elderly PC patients undergoing surgical treatment. In addition, radiotherapy, chemotherapy, and marriage also have some effects.

**Figure 3:**
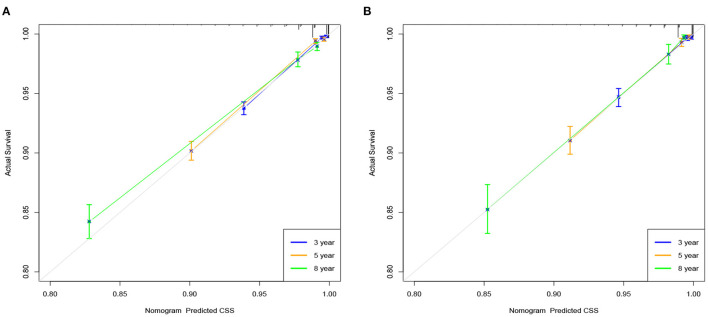
Calibration curve of the nomogram in the training set **(A)** and validation set **(B)**. The horizontal axis is the predicted value in the nomogram, and the vertical axis is the observed value.

**Figure 4:**
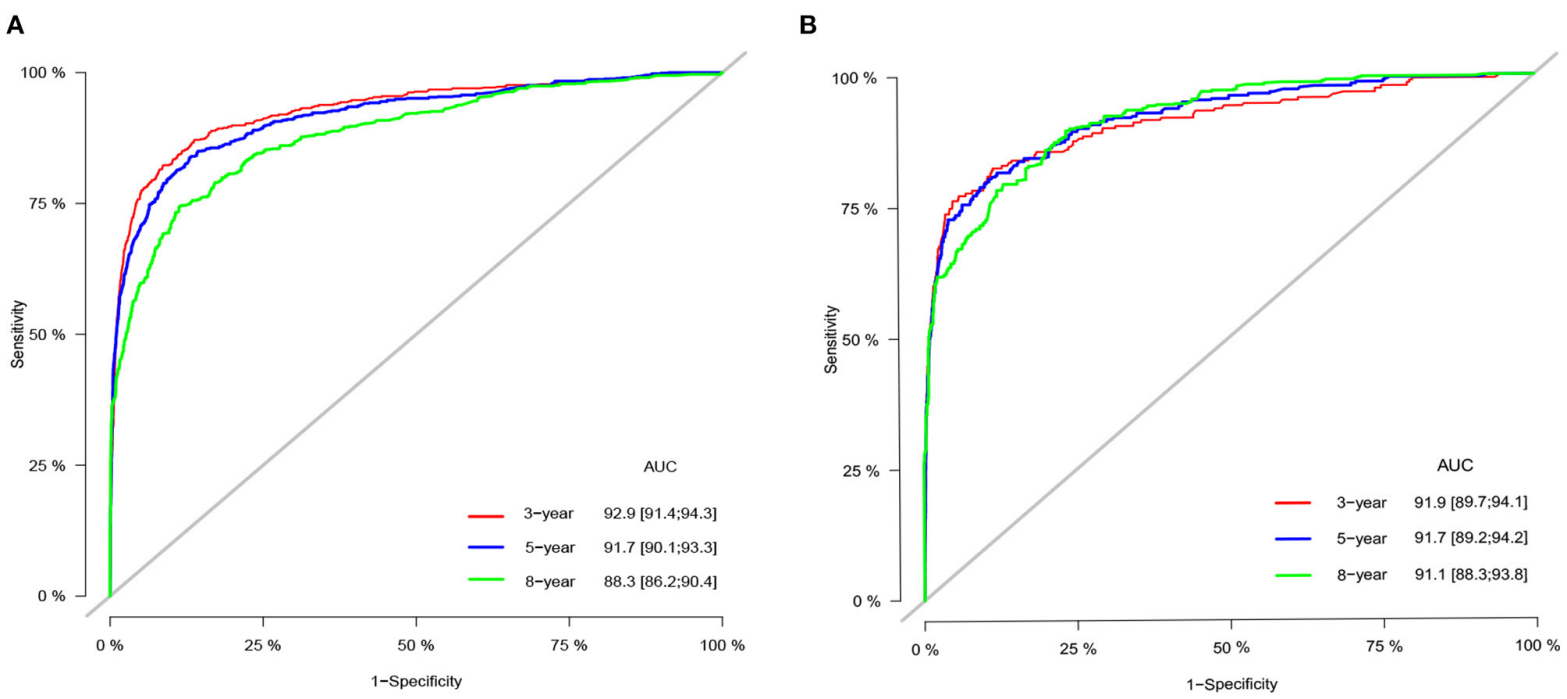
AUC for predicting 3-, 5-, and 8-year CSS in the training set **(A)** and validation set **(B)**.

We used internal cross-validation to validate the model's accuracy and discriminability. The C-index for the training and validation sets is 0.911(95%CI: 0.899–0.923) and 0.913(95%CI: 0.893–0.933, respectively), indicating that the prediction model of the nomogram has a good recognition ability. The calibration curves of the training and validation sets show that the predicted values of the nomogram are highly consistent with the actual observations (Figure 3), indicating that the nomogram has good accuracy. AUC was used to detect the discrimination of the nomogram, AUC at 3-,5-and 8-year was 92.9, 91.7, and 88.3 in the training set, and in the validation set, AUC at 3-,5-, and 8-year was 91.9,91.1 and 91.7, respectively (Figure 4).

### Clinical Application of the Nomogram

**Figure 5:**
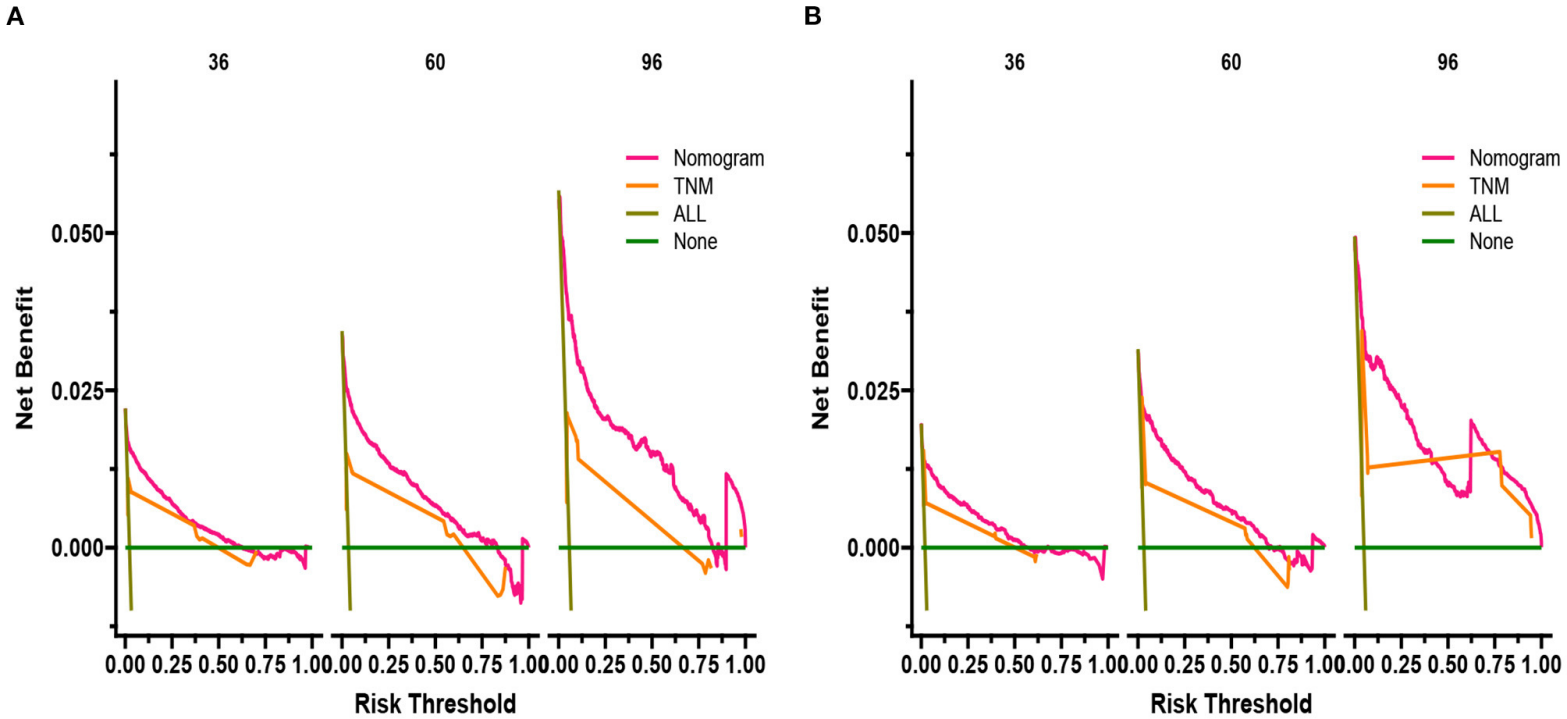
DCA of the nomogram in the training set **(A)** and the validation set **(B)**. The Y-axis represents a net benefit, and the X-axis represents threshold probability. The green line means no patients died, and the dark green line means all patients died. When the threshold probability is between 0% and 100%, the net benefit of the model exceeds all deaths or none.

**Figure 6:**
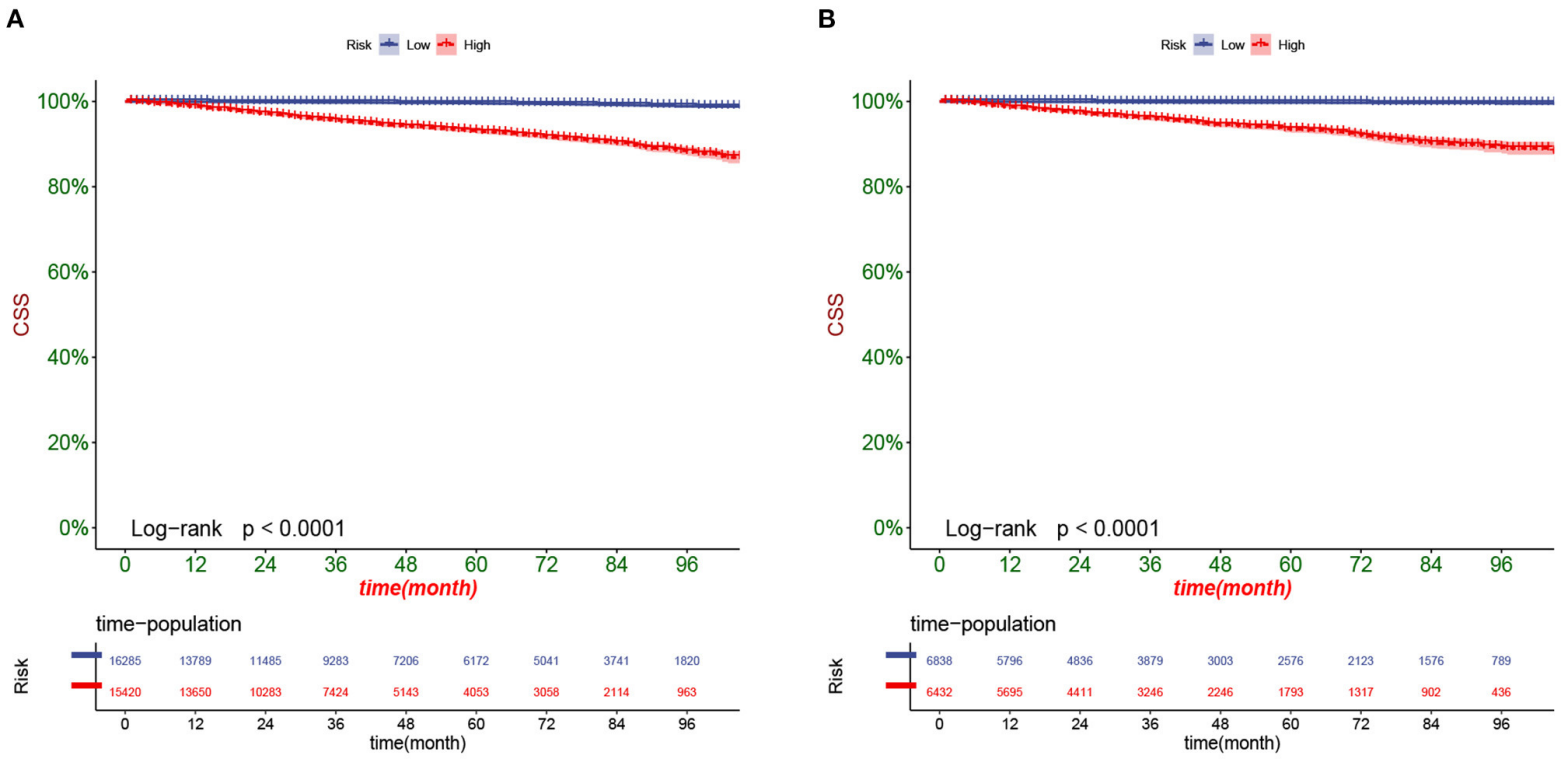
Kaplan-Meier curves of patients in the low-risk and high-risk groups in the training set **(A)** and validation set **(B)**.

**Figure 7:**
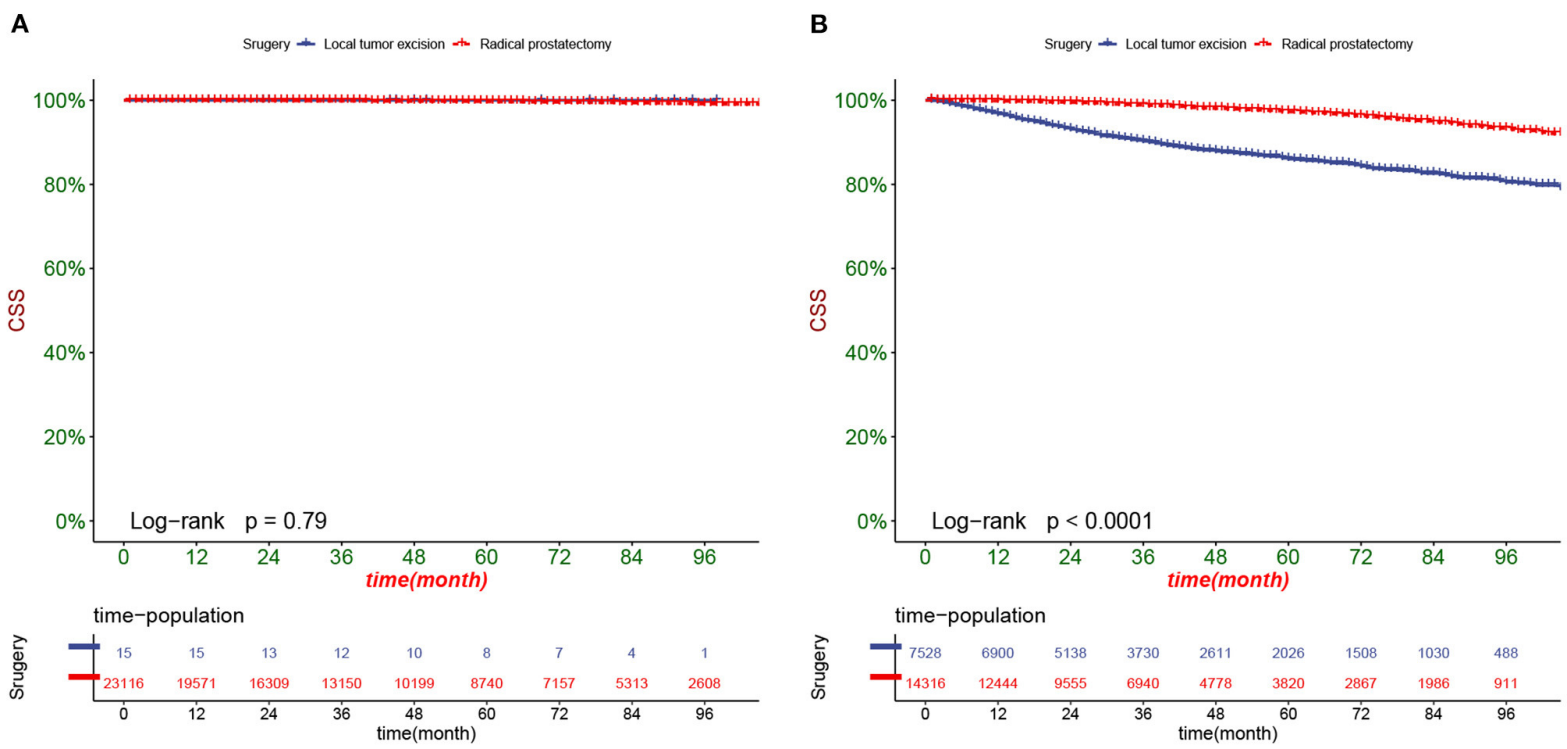
Kaplan-Meier curves of patients with different surgery in the low-risk group **(A)** and high-risk group **(B)**.

**Figure 8:**
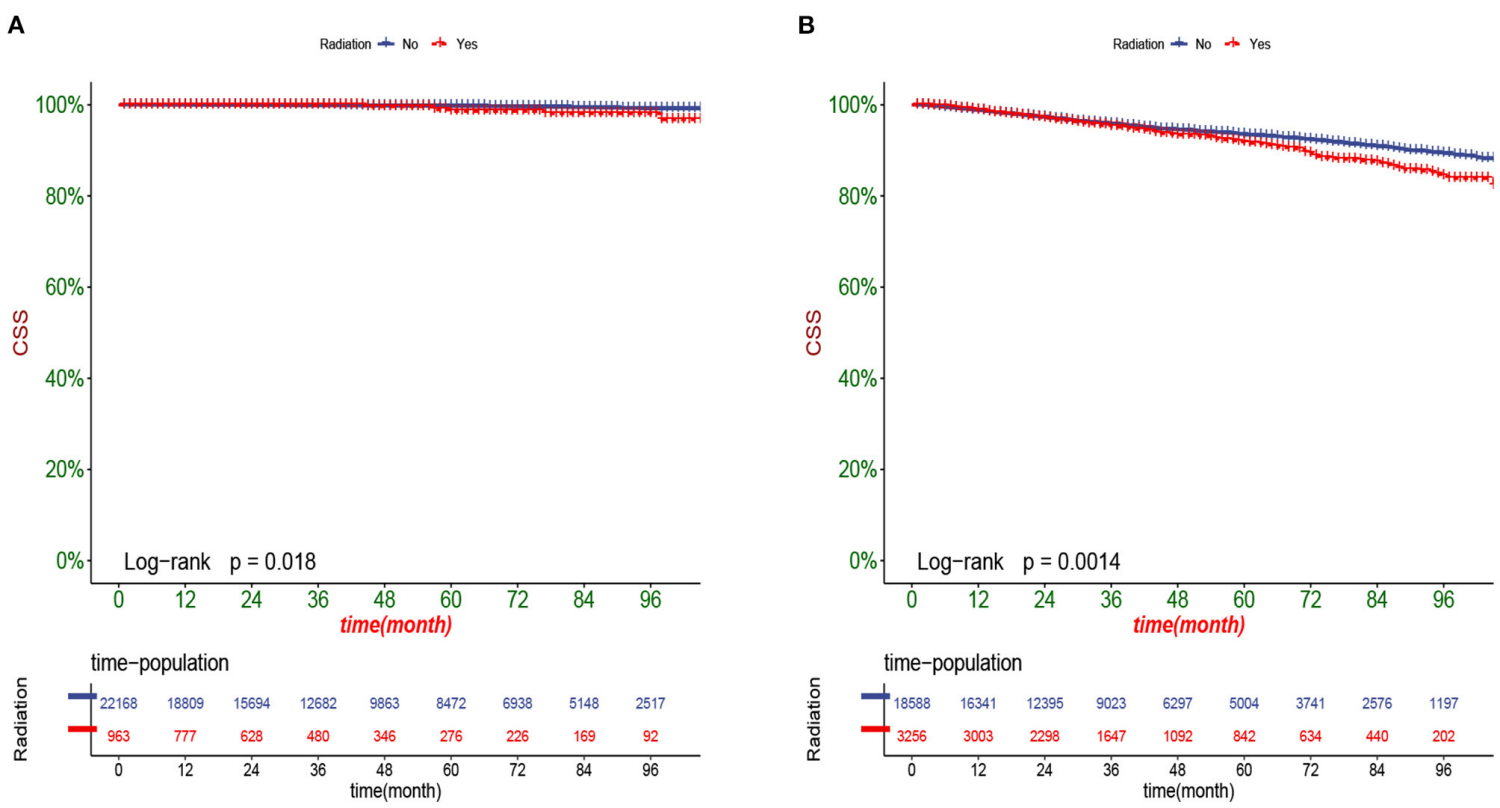
Kaplan-Meier curves of patients with or without radiotherapy in the low-risk group **(A)** and high-risk group **(B)**.

DCA responds to the clinical value of the nomogram, and our results showed that it has good clinical potential value in both the training group and the validation set (Figure 5). Furthermore, we calculated the risk value for each patient based on the nomogram and calculated the optimal cutoff value using the ROC curve. Patients were classified into high-risk (total score 114.33) and low-risk (total score <114.33). The K-M curve showed that the survival rate of patients in the low-risk group was significantly higher than that in both the high-risk group and the high-risk group (Figure 6). The high-risk group of patients with 3-, 5-, and 8-year survival rates were 97.7%, 93.1%, and 88.6%, respectively. The low-risk group's 3-, 5- and 8-year survival rates were 99.8%, 99.6%, and 99.1%, respectively. Although most of the high-risk group patients underwent radical prostatectomy, the patients undergoing local tumor resection had higher survival rates. Almost all patients in the low-risk group underwent radical prostatectomy, so there was no significant difference in patient survival (Figure 7). We found that the survival rate of patients in the high-risk group receiving radiotherapy was lower than those who did not, considering that most older patients in the high-risk group had comorbidities and side effects of radiotherapy. In contrast, the survival rate of patients receiving and without radiotherapy in the low-risk group was not significantly different (Figure 8).

## Discussion

Although the traditional TNM stage is widely used in cancer management, multiple factors affect the prognosis clinically, and the TNM stage does not include these clinical factors. The prostate-specific antigen and Gleason score are critical factors for the prognosis of PC patients. Therefore, the most commonly used risk stratification scheme, D'Amico, combines clinical-stage, prostate-specific antigen, and Gleason score (16). Patients were divided into three risk levels: low risk (clinical stage T1-T2a, prostate-specific antigen ≤ 10 ng/mL and Gleason score ≤ 6), medium risk (T2b or <10 ≤ prostate-specifc antigen 20 ng/mL or Gleason score 7), or high-risk disease (stage T2c or prostate-specific antigen> 20 ng/mL or Gleason score≥8). Although the D'Amico risk assessment protocol included prostate-specific antigen and Gleason score compared with the TNM stage, it still ignores the impact of age, ethnicity, marriage, surgical style, and chemoradiotherapy on the survival of PC patients.

The SEER database contains data from 18 medical centers analyzing prominent samples representative of populations from different regions. The SEER database contains patient demographic characteristics (age, race, marital status), tumor grade, TNM stage, treatment mode, and prognostic information, including survival status, cause of death, and survival time. The nomogram is a convenient and reliable statistical prediction tool that also uses multiple clinical variables different from the TNM stage to predict the prognosis of cancer patients (17). The nomogram can provide an individualized prediction of CSS through a comprehensive analysis of these clinicopathological factors. Nomogram meets the requirements of the integrated model. It promotes personalized medicine to facilitate clinicians' use for prognosis prediction using the SEER database. Previous virus studies have developed nomograms for predicting the OS and CSS of multiple databases. Moreover, it has shown promising clinical potential value in renal cancer, endometrial stromal sarcoma, glioma, nephroblastoma, and other cancers (18–20).

It is well known that the chance of genetic mutations inducing cancer increases with age. Studies have shown that age has a crucial role in the survival rate of various cancers (21). PC is no exception, and its incidence was significantly associated with age. PC was more frequent in older men, and the median age of diagnosis was 66 years (22). Multiple predictive models for the PC also showed that age is an independent risk factor for the prognosis of PC patients (15). Our results also show that age is an essential factor affecting patient prognosis. In addition, we found that age was linearly associated with poorer prognostic outcomes in elderly patients older than 65 years, with no significant cutoff value.

It has been reported in much literature that marriage benefits the prognosis of most cancer patients due to more social and financial support after marriage, as well as partner psychological comfort to patients (23). Our results show that marriage is also a protective factor for elderly PC patients undergoing surgery. Previous literature has reported the prevalence of PC among different ethnic groups, and the prognosis was also significantly associated with race (24). However, our study found that race was not an independent risk factor for elderly PC patients undergoing surgery. We considered that this might be related to differences in financial ability and medical level among different ethnic groups, leading to selection bias in treatment modalities.

Prostate-specific antigen screening began in the late 1980s, leading to a sharp increase in PC incidence over a short period (25). In 2012, the US Preventive Services Working Group (USPSTF) reported that prostate-specific antigen screening reduced the risk of PC death, and the USPSTF recommended including prostate-specific antigen in PC screening (26). The impact of screen-detected active treatment for PC on long-term survival compared to active surveillance is unknown but is associated with sexual dysfunction and dysuria (27). A secondary analysis of two randomized clinical trials by Martin et al. showed that increased prostate-specific antigen levels after treatment might be associated with a worse prognosis for locally advanced PC in men (28). The D'Amico risk assessment scheme divided prostate-specific antigen into three grades, prostate-specific antigen ≤10 ng/mL, 10–20 ng/mL, > 20 ng/mL. Our prediction model also divided prostate-specific antigen according to this standard. The results showed that the higher the prostate-specific antigen expression level, the worse the patient prognosis, consistent with previous studies (29). Meanwhile, the Gleason score serves as an important tool for predicting the prognosis of PC patients. Our prediction model also divides it into three grades according to the D Amico protocol, Gleason score≤6, Gleason score 7, and Gleason score≥8. Our results showed that the Gleason score of most elderly PC patients undergoing surgical treatment was 7, the worst prognosis for Gleason score≥8, and the best prognosis for Gleason score≤6, which is also supported by previous studies (30, 31).

PC treatment mainly includes active surveillance, surgical resection, radiotherapy, chemotherapy, ADT, and other comprehensive treatment methods. Surgical resection mainly includes radical prostatectomy and local tumor resection. Radiotherapy and radical prostatectomy are evidence-based treatments for non-metastatic PC and can reduce PC mortality compared with non-therapeutic randomized clinical trials (32). A ProtecT randomized controlled trial by David E Neal et al. showed that 90% of localized patients did not die of PC within 10 years. Whether through active surveillance, surgery, or radiotherapy, active surveillance had fewer side effects on sexual and bladder function but a greater risk of tumor spread (33). The study by Freddie C Hamdy et al. confirmed that surgery and radiotherapy were associated with a lower incidence of disease progression and metastasis than active surveillance (34). A randomized trial conducted by Lars Holmberg et al. showed that radical prostatectomy significantly reduced CSS in early-stage PC patients but showed no significant difference between surgery and observation waiting for OS (40). Anna Bill-Axelson et al. confirmed that radical prostatectomy was associated with reduced mortality in PC, but adjuvant local or systemic therapy may be more beneficial in men with extracapsular tumor growth; they also concluded that radical prostatectomy showed no benefit in elderly PC patients over 65 years (35). After nearly 20 years of follow-up of men with localized PC, Timothy J Wilt et al. found that surgery was not associated with significantly reduced mortality and a higher frequency of adverse events than active surveillance (36). The above studies show that different treatment modalities' outcomes differ significantly for different types of PC patients. Our results showed that radical prostatectomy is associated with a better prognosis than local tumor resection; postoperative radiotherapy achieves a better prognosis than patients without radiotherapy. However, our nomogram shows that chemotherapy is associated with a worse prognosis. We consider why patients receiving chemotherapy often relapsed or have distant metastasis and have a worse prognosis. At the same time, the small number of people receiving chemotherapy results in biased results.

The nomogram based on the SEER database has good accuracy, but this study has some shortcomings. First, there is a lack of essential information, such as ADT, which is one of the primary treatment modalities for PC; however, there is a lack of data related to ADT in the SEER database, so our model also lacks the relationship between ADT and prognosis. Moreover, PSA is an important indicator related to prognosis for PC patients. However, it was not included in the SEER database until 2010, so we can only choose data after 2010 to build a predictive model, resulting in a smaller sample size for us to choose. Second, our nomogram could not include all related prognostic variables such as BMI, smoking, alcohol consumption, Etc. leading to some limitations in our results. Finally, our study was retrospective, which may lead to an unavoidable risk of selection bias. Although our results have some limitations, our nomogram still includes key prognostic variables and has been internally validated to show good accuracy, so the results are not significantly biased. Second, our nomogram shows good potential clinical application value, which can help doctors and patients to make clinical decision-making.

## Conclusion

Our study explored the clinicopathological factors affecting CSS in elderly PC patients undergoing surgical treatment. We found that age, marriage, prostate-specific antigen, Gleason score, surgical mode, chemoradiotherapy, and TNM stage were independent risk factors affecting CSS in patients. We established a nomogram that could predict the CSS in elderly PC patients undergoing surgical treatment. The model has been internally validated with good accuracy and reliability. It showed better potential clinical application value than the TNM staging system; it can help clinicians and patients make clinical decisions. However, our study is retrospective, which may lead to an unavoidable risk of selection bias. Future prospective studies with large and multicenter samples are needed to validate the nomogram further.

## Data Availability Statement

The datasets presented in this study can be found in online repositories. The names of the repository/repositories and accession number(s) can be found in the article/Supplementary Material.

## Author Contributions

ZZ and CZ designed the study. ZZ, JW, JL, LJ, and XT collected and analyzed the data. ZZ drafted the initial manuscript. CZ, TM, JL, and DH revised the article critically. CZ, JL, and DH reviewed and edited the article. All authors approved the final manuscript.

## Funding

Special Key Project of Chongqing Technology Innovation and Application Development (No. Cstc2019jscx-tjsbX0003), Yunnan Education Department of Science Research Fund (No. 2020 J0228), Kunming City Health Science and Technology Talent 1000 training Project (No. 2020- SW (Reserve)-112), Kunming Health and Health Commission Health Research Project (No. 2020-0201-001), and Kunming Medical Joint Project of Yunnan Science and Technology Department (No. 202001 AY070001-271).

## Conflict of Interest

The authors declare that the research was conducted in the absence of any commercial or financial relationships that could be construed as a potential conflict of interest.

## Publisher's Note

All claims expressed in this article are solely those of the authors and do not necessarily represent those of their affiliated organizations, or those of the publisher, the editors and the reviewers. Any product that may be evaluated in this article, or claim that may be made by its manufacturer, is not guaranteed or endorsed by the publisher.
